# A Case of Adipophilin-Positive Pancreatic Intraductal Papillary Mucinous Neoplasm (IPMN) With Eosinophilia

**DOI:** 10.7759/cureus.93715

**Published:** 2025-10-02

**Authors:** Takahiro Sato, Yo Kato

**Affiliations:** 1 Department of Gastroenterology, Honjo-Fukushima Hospital, Honjo, JPN; 2 Department of Surgery, Nippon Medical School, Bunkyo-ku, JPN; 3 Departments of Pathology, Cancer Institute Hospital of the Japanese Foundation for Cancer Research, Koto-ku, JPN

**Keywords:** adipophilin, branch duct type, eosinophilia, gastric type, intraductal papillary mucinous neoplasm

## Abstract

Adipophilin expression and eosinophilia associated with intraductal papillary mucinous neoplasm (IPMN) are extremely rare in the literature, and the relationship between adipophilin expression and IPMN remains unclear. This study reports the case of a 67-year-old male patient with adipophilin-positive IPMN and eosinophilia, which diminished following R0 surgical resection. The patient underwent pancreaticoduodenectomy for a pancreatic head (Ph) tumor detected by abdominal echo screening. Computed tomography revealed a polycystic mass approximately 40 mm in diameter. The resected specimen was located in the Ph and measured 35 × 31 × 20 mm (TS2). Histopathological examination revealed an IPMN, low-grade papillary growth, and a branch duct type. Immunohistochemically, mucin 5AC (MUC5AC) and MUC6 were expressed in the tumor cytoplasm, classifying it as the gastric type. In addition, eosinophil infiltration was observed in the glandular ducts of the tumor, and adipophilin was expressed in the tumor cytoplasm. This case highlights the rare coexistence of adipophilin expression and eosinophilia in IPMN, necessitating further investigation.

## Introduction

Intraductal papillary mucinous neoplasm (IPMN) is incidentally detected by diagnostic imaging such as abdominal ultrasound and computed tomography (CT) without symptoms. On the other hand, it is thought to be the background of malignant progression to pancreatic adenocarcinoma. Moreover, this lesion is classified according to morphological and histopathological grades [1,2]. Following the guidelines of IPMN, close sequential surveillance is necessary for the decision of appropriate treatment. Reports of eosinophilia, refractory skin rash, and adipophilin associated with IPMN are extremely rare in the literature [1,2,3,4,5]. Adipophilin (ADPH, ADFP), also known as adipose differentiation-related protein (ADRP) and perilipin 2, is a lipid droplet membrane protein belonging to the PAT family (perilipin, adipophilin, and tail-interacting protein of 47 kDa (TIP47), etc.). It is pivotal in fat storage, degradation, and protection. Adipophilin is widely recognized as a highly useful marker for lipid droplets, with membrane positivity indicating the presence of intracytoplasmic lipid droplets [6]. In addition, adipophilin is crucial in fatty acid metabolism and insulin secretion by pancreatic islets [7]. Recently, apolipoprotein A2 isoform-i (apoA2-i), a lipid metabolism marker, has been identified as a biomarker for high-risk individuals (HRIs) [8]. Although eosinophilia is induced by various factors, the association between adipophilin expression and eosinophilia in IPMN is rare. We report a case where eosinophilia significantly decreased in the short term following radical resection of adipophilin-positive IPMN.

## Case presentation

**Figure 1:**
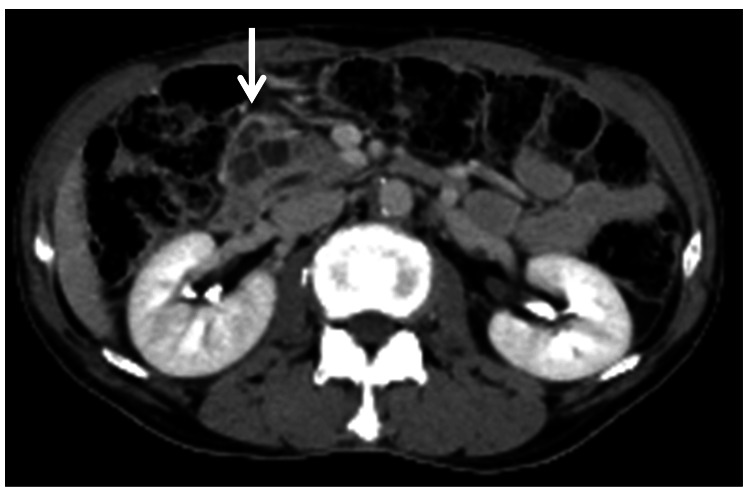
Contrast-enhanced CT revealing a polycystic mass with a diameter of about 40 mm

A 67-year-old male was referred to the Satellite Hospital of Nippon Medical School (Tokyo, Japan) for evaluation of a pancreatic head (Ph) tumor detected via abdominal ultrasound. CT revealed a polycystic mass approximately 40 mm in diameter (Fig. 1). 

The patient had been experiencing itching and partially painful rashes all over the body for approximately six months. He had previously been diagnosed with nonspecific pruritus at the local dermatology clinic.

His medical history included diabetes mellitus, which was well controlled with medication. However, physical examination did not reveal a palpable mass. No anemia or jaundice was observed in the eyelid conjunctiva or ocular conjunctiva, respectively.

Preoperative blood examination revealed an eosinophil count of 1287.4/μL and an increased carcinoembryonic antigen of 9.0 ng/mL. No other abnormalities were observed. The tumor was diagnosed as Ph IPMN, which met the relative surgical criteria of the IPMN guidelines [1,2]. The patient underwent a pancreaticoduodenectomy. The surgery lasted 359 minutes, with 950 g of blood loss. The patient was discharged uneventfully 35 days after surgery.

**Figure 2:**
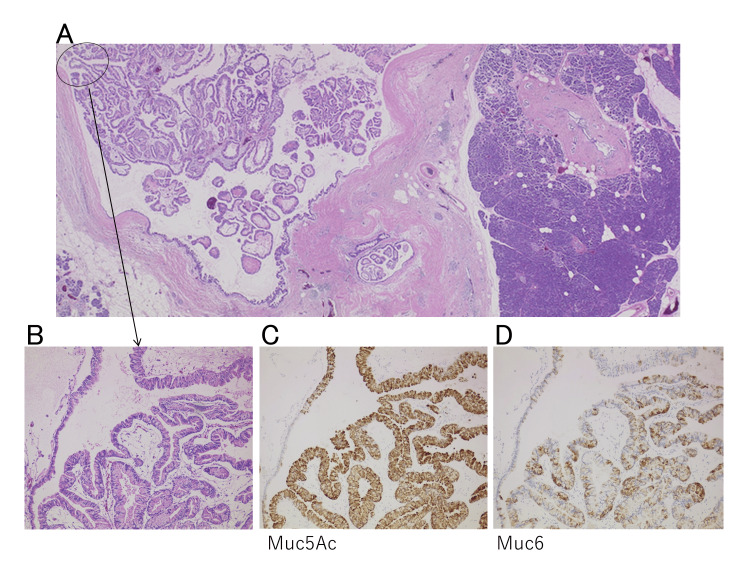
Pathological findings (A) In weak magnification, IPMN was low-grade with no malignant findings. (BD: branch duct type) (B) In medium magnification, the main lesion (intraductal papillary mucinous neoplasm, low-grade) exhibits papillary growth. (C) In medium magnification, MUC5AC is immuhistochemically positive. (D) In medium magnification, MUC6 is immuhistochemically positive. This IPMN is gastric type.

The resected specimens included the Ph, TS2, and a mass measuring 35 × 31 × 20 mm. Histopathological examination revealed an IPMN, low-grade papillary growth, and branch duct type (BD). Immunohistochemical analysis showed positivity for Mucin 5AC (MUC5AC) and MUC6, classifying the tumor as a gastric-type IPMN (Fig. 2).

**Figure 3:**
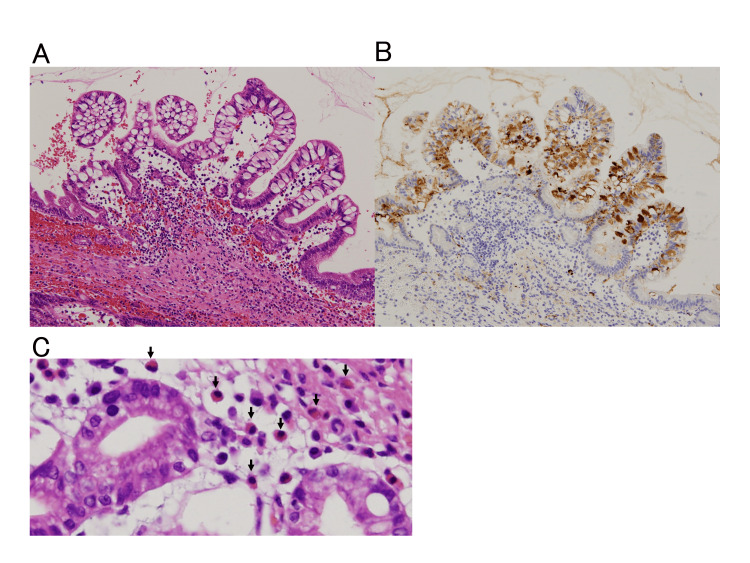
Eosinophils, adipophilin, and intraductal papillary mucinous neoplasm (IPMN) (A) Medium magnification: Eosinophils infiltrate around the tumor ducts. (B) Medium magnification, immunostaining: Adipophilin is expressed in the tumor cytoplasm. (C) Strong magnification: Eosinophils (arrows) infiltrate around the tumor ducts.

Eosinophils infiltrated the glandular ducts of the tumor, and adipophilin was expressed in the cytoplasm of the gland ducts (Fig. 3).

After surgery, itching, skin redness, and blisters resolved by the second week. By the third week, the skin lesions had reduced to pigmentation. Simultaneously, the eosinophil count decreased to 296.7/μL.

## Discussion

This is the first report to perform an immunohistochemical analysis of adipophilin in IPMN. Adipophilin, an antibody that recognizes proteins associated with lipid droplets, serves as an immunohistochemical marker [6]. This suggests that IPMN may contain lipid droplets within the cytoplasm, despite the presence of xanthomatous differentiation on light microscopy. Animal studies indicate that concurrent G-protein αs (GNAS) and KRAS mutations characterize pancreatic ductal adenocarcinomas (PDAs) arising from IPMNs, associated with the induction of lipid remodeling and fatty acid oxidation [9].

Integrated metabolomic and lipidomic analyses of plasma or cyst fluid can discriminate between IPMN and serous cystic neoplasm (sCN) and also predict IPMN dysplasia grade [10]. This study reports the case of a patient with adipophilin-positive IPMN and eosinophilia, which resolved following R0 surgical resection. Reports of refractory skin rashes associated with IPMN are rare. In this study, eosinophilia and skin rash subsided after radical resection. Additionally, adipophilin was expressed in the tumor cells of the resected pancreas, and eosinophils were observed around the tumor cells. Eosinophilia was defined as a peripheral blood eosinophil count > 500/μL (0.5 × 109/L). Numerous etiologies, primarily allergic reactions and parasitic infections, have been associated with eosinophilia [11], which is occasionally induced by necrosis [12]. Interleukin (IL)-5 is one of the cytokines involved in the proliferation, maturation, activation, and recruitment of eosinophils [13]. While most studies focus on protein mediators such as interleukin (IL)-5 and eotaxins, lipid mediators also influence eosinophil recruitment and activation [14]. Increasing evidence suggests that eosinophil accumulation induced by lipopolysaccharide (LPS) arises from eosinophil-lactic cytokine produced through macrophage and T-cell interactions at the site of an LPS-induced inflammatory reaction [15]. However, no infections were observed in our case study. This case was classified as mild eosinophilia, and no such history was observed; the eosinophil accumulation was likely due to adipophilin deposition. Although cystic fluid analysis of pancreatic IPMN was not performed, cytokines in the pancreatic cystic fluid may have contributed to eosinophilia. Pancreatic cyst fluid prostaglandin E2 (PGE2) is an indicator of IPMN dysplasia [16]. Combining cyst fluid PGE2, IL-1β, and serum CA 19-9 optimizes specificity and positive predictive value (PPV) for high-grade dysplasia (HGD)/invasive IPMN [17]. Some studies have reported a close relationship between eosinophils and pancreatic tumors.

According to immunological animal experiments, M2 macrophages and eosinophils co-localized with fibrotic regions rather than with infiltrating tumors, consistent with immune cell privilege. Most eosinophils in the pancreas of Akt1Myr/KRasG12D mice with chronic inflammation lacked cytotoxic NKG2D markers. IL-5 expression is upregulated in pancreatic cells in response to inflammation and diminishes in advanced lesions [18]. The secreted enzyme autotaxin (ATX) hydrolyzes extracellular lysophosphatidylcholine to generate the multifunctional lipid mediator lysophosphatidic acid (LPA) and supports tumor growth, including PDAC. Eosinophils have been identified in human PDAC specimens, and rare cases with high intratumoral eosinophil abundance exhibit the longest overall survival. These findings suggest a context-dependent immunomodulatory role of ATX-LPA signaling in cancer [19].

Although a direct relationship between adipophilin and eosinophils is unlikely, adipophilin was expressed in the cytoplasm of tumors. Considering the resolution of redness and blisters after complete resection of the tumor, it is suspected that adipophilin may have caused eosinophilia, although the mechanism remains unknown. A correlation has been observed between adipophilin expression and malignancy in salivary gland carcinoma [20]. While tumors in this case lacked carcinomatous or dysplastic components, careful postoperative follow-up is essential. 

## Conclusions

This study reports the case of a 67-year-old male patient with adipophilin-positive IPMN and eosinophilia, which diminished following R0 surgical resection. This case study highlights two key findings: (1) adipophilin expression in the tumor cytoplasm of IPMN and (2) resolution of eosinophilia after R0 resection. The presence of eosinophilic infiltration near adipophilin-positive tumor cells suggests a role for cytokines and lipid metabolism in eosinophil recruitment. We hypothesize that this case study will contribute to the understanding of the malignant progression pathway of IPMN. Of course, due to certain limitations of this case study, further large-scale studies are needed.
